# Supplementary material for: Therapeutic relationships within child and adolescent mental health inpatient services: A qualitative exploration of the experiences of young people, family members and nursing staff
Source: PLoS One. 2022 Jan 14;17(1):e0262070. doi: 10.1371/journal.pone.0262070 (PMC8759657; doi:10.1371/journal.pone.0262070)
Supplement: Attachment — Submitted filename: Response to reviewers.docx [file pone.0262070.s002.docx]

**Therapeutic relationships within child and adolescent mental health inpatient services: a qualitative exploration of the experiences of young people, family members and nursing staff**

Dear Dr O’Connor, Thank you for your prior review and current support as editor.

We welcome the thoughtful suggestions and have responded to each of these below.

We hope you will agree that the changes have improved the paper and it is now of a quality suitable for publishing in PLOS One.

- **Line 29-32 – the punctuation in this sentence is confusing. It would be helpful to demarcate the theme titles with capitalisation, quote marks or italics (similarly in line 214-216)**

We have now italicised and capitalised the theme names in both sections.

- **Line 49 – should not start a sentence with ‘But’**

This has now been amended:

*“However, questions remain: what is ‘high quality’ and what do young people need to support their care in these environments?”*

- **Line 121 – state the official name of the ethics committee that approved the research**

This has now been added: *“(West of Scotland REC 4; IRAS ID 246547).”*

- **Line 180-183 – inconsistent capitalisation of ‘author’**

This has been consistently changed to “author”.

- **Line 187 – “omitting data that was not relevant to the research question or not related to nursing staff” – this is not clear as you have not explicitly stated a specific research question, and there is no indication of what is meant by ‘not related to nursing staff’ given that not all participants were nurses**

This has now been clarified: “omitting data that was not relevant to therapeutic relationships between nursing staff and young people/family members”

- **Line 216 – capitalise Fig 2**

This has been capitalised.

- **Line 643, 735, 859 – avoid contractions (unless a direct quote)**
- **Lines 653-654, 724 – inappropriate apostrophes**

These are all direct quotes from transcripts and reflect the voice of the participants.

- **Please ensure consistency throughout the Results section regarding whether quotes on new lines are preceded by a colon or semi-colon.**

This has been standardised to use colons throughout.

- **Minor typos/errors in lines 429, 552, 562, 658, 661, 673, 764, 814, 859**

Thank you; these have been corrected.

**Additionally, certain reviewer comments have not been directly addressed within the revised manuscript. Specifically:**

- **R1’s query about the relationships between participants should be clarified within the Sample section**

Detail on this has been added to the sampling section:

*“In terms of connections between participants, we neither encouraged nor prevented participants from the same family taking part, which is an approach that was approved by our ethics committee review. In terms of staffing relationships, participants were recruited across four sites and some staff will have cared for the young people interviewed. The nature of inpatient care is that all staff members will have contact/ relationships of varying degrees with all young people and family members on the unit.”*

- **R2’s request for quotes to be accompanied by participant ID numbers is appropriate and consistent with COREQ guidelines and PLOS ONE norms for qualitative articles**

ID numbers have now been added throughout.

- **R3’s request for clarification of the quote about the ‘egg’ is appropriate – the meaning of this quote is not accessible to a reader without the type of contextualisation provided in the response to reviewers**

The explanation given in our previous response to reviewers has now been added to the body of the paper:

*“Here, the staff member describes how when boiling an egg in its shell if you remove it from the water, the surface dries immediately. Therefore, this idiom illustrates that staff need to let things wash over them quickly – be impervious to emotional distress. Another version might be ‘like water off a duck’s back’. In reality, this cannot always be possible….”*
